# An adaptive two-arm clinical trial using early endpoints to inform decision making: design for a study of sub-acromial spacers for repair of rotator cuff tendon tears

**DOI:** 10.1186/s13063-019-3708-6

**Published:** 2019-12-09

**Authors:** Nick Parsons, Nigel Stallard, Helen Parsons, Philip Wells, Martin Underwood, James Mason, Andrew Metcalfe

**Affiliations:** 1 Statistics and Epidemiology Unit, Warwick Medical School, University of Warwick, Coventry, CV4 7AL UK; 2 Warwick Clinical Trials Unit, Warwick Medical School, University of Warwick, Coventry, CV4 7AL UK; 3 University Hospital Coventry and Warwickshire, Coventry, CV2 2DX UK

**Keywords:** Adaptive design, Stopping for futility, Early endpoints

## Abstract

**Background:**

There is widespread concern across the clinical and research communities that clinical trials, powered for patient-reported outcomes, testing new surgical procedures are often expensive and time-consuming, particularly when the new intervention is shown to be no better than the standard. Conventional (non-adaptive) randomised controlled trials (RCTs) are perceived as being particularly inefficient in this setting. Therefore, we have developed an adaptive group sequential design that allows early endpoints to inform decision making and show, through simulations and a worked example, that these designs are feasible and often preferable to conventional non-adaptive designs. The methodology is motivated by an ongoing clinical trial investigating a saline-filled balloon, inserted above the main joint of the shoulder at the end of arthroscopic debridement, for treatment of tears of rotor cuff tendons. This research question and setting is typical of many studies undertaken to assess new surgical procedures.

**Methods:**

Test statistics are presented based on the setting of two early outcomes, and methods for estimation of sequential stopping boundaries are described. A framework for the implementation of simulations to evaluate design characteristics is also described.

**Results:**

Simulations show that designs with one, two and three early looks are feasible and, with appropriately chosen futility stopping boundaries, have appealing design characteristics. A number of possible design options are described that have good power and a high probability of stopping for futility if there is no evidence of a treatment effect at early looks. A worked example, with code in R, provides a practical demonstration of how the design might work in a real study.

**Conclusions:**

In summary, we show that adaptive designs are feasible and could work in practice. We describe the operating characteristics of the designs and provide guidelines for appropriate values for the stopping boundaries for the START:REACTS (Sub-acromial spacer for Tears Affecting Rotator cuff Tendons: a Randomised, Efficient, Adaptive Clinical Trial in Surgery) study.

**Trial registration:**

ISRCTN Registry, ISRCTN17825590. Registered on 5 March 2018.

## Background

New surgical procedures are usually introduced based on what a surgeon believes might benefit patients and nothing more. Whilst pharmaceuticals undergo rigorous clinical trials before introduction, this is not the case for surgical procedures, which are often introduced based purely on basic science (such as cadaveric testing) or small case series data only. There is a need to develop new processes and methodology to introduce surgical procedures safely [1–3], with early randomised controlled trials (RCTs) in specialist centres used to determine whether a treatment is likely to be safe, clinically effective and cost effective prior to widespread uptake. Large clinical trials powered for patient-reported outcomes are typically expensive and often take more than 5 years from award to completion. Ineffective, unsafe and costly treatments may be used for many years before they are removed from practice. This is clearly unacceptable and unethical. Conversely, very effective treatments may be withheld from widespread practice until trials are complete, leading to long delays in the delivery of worthwhile treatments for patients. Trial designs are required which can efficiently and rapidly determine that a procedure is ineffective or harmful, but will also adapt to demonstrate superiority if the technique is a genuine improvement on standard care. There is a growing awareness amongst both funders and researchers that conventional clinical trial designs are not the best option in many settings, and that novel adaptive design methods offer the potential to undertake clinical trials in a much more flexible manner, whilst retaining trial integrity.

An adaptive clinical trial allows for prospectively planned changes to be made to some aspects of the design as it proceeds, using data collected from participants recruited into the study. These types of designs have grown in popularity in recent years [4], providing flexibility for trialists to, for instance, refine sample sizes, drop interventions (or doses of a drug), identify and focus recruitment on responsive subgroups (enrichment) or stop studies early [5]. For trials of new surgical interventions, the option to potentially stop the study early has particular appeal. The advantages of stopping a trial early are twofold. First, in many widely encountered settings it is likely to make the trial design more efficient [5, 6]. For instance, if a test treatment is in truth much less effective than initially anticipated (or is totally ineffective), then the expected sample size and duration of a design that allows early stopping will be less than those of a comparable conventional fixed sample size (non-adaptive) design. Second, stopping a study early because an intervention is shown to be ineffective (under the null hypothesis) or conversely is shown to be effective (under the alternative) is clearly ethically beneficial, as it allows people to receive better treatments faster. Adaptive designs offer the potential of considerable advantages when compared to more conventional fixed designs; however, there are often barriers to their implementation [7] and disadvantages, such as the requirement to use or develop more complex statistical tools, the additional pressures on data monitoring and collection and the maintenance of trial integrity [8].

In surgical trials, participants are often routinely followed up at a number of occasions (e.g. 3, 6 and 12 months) and the main study outcome(s) are collected at each occasion. Therefore, at an interim analysis there will be some participants with 3-month data, some with 3- and 6-month data and some with 3-, 6- and 12-month data. If interim analyses are limited to only those participants with 12-month data (primary outcome), then the opportunities for early stopping if there is evidence to support either treatment *futility* or *efficacy* may well be severely limited due to time constraints; i.e. recruitment may well have completed before enough 12-month outcome data are available for reliable decision making. If early endpoints are correlated with the definitive (final) study endpoint, then clearly an analysis that ignores the early endpoints for interim decision making is likely to be inefficient. Stallard [9] showed that using *short-term* (or what others often call *early endpoint*) data, in the setting of a seamless phase II/III clinical trial with treatment selection with a single early endpoint, leads to increases in statistical power when these data are correlated with the primary endpoint.

As a consequence of the perceived lack of efficiency and inflexibility of traditional RCTs, the UK National Institute for Health Research (NIHR) [10] is funding a surgical RCT that will use a novel adaptive study design approach, developed specifically for the evaluation of new surgical procedures (Efficacy and Mechanism Evaluation Programme: 16/61 Evaluation of new surgical procedures through the use of novel study designs). This RCT provides the motivation for the work outlined here. In this paper we adapt the approach previously described by Stallard [9], which used a single early endpoint in a treatment selection design. Here we generalise to the setting with more than one early endpoint for comparing two treatment groups [11], and outline how the methodology can be used for interim decision making using an ongoing study of sub-acromial spacers for rotator cuff tendon tears as an exemplar. We start by providing the clinical context and then develop a model for the distribution of the outcomes, and give an expression for an appropriate test statistic and describe how inferences and decisions about stopping are made in the chosen setting. Simulations are undertaken and operating characteristics are illustrated for a wide range of design options. The aim of the work described here is to outline the process undertaken to develop a design for the specific trial that motivated this work. The final selection of the design options for that study will be made by and remain confidential within the study team. A practical worked example, using synthetic data, is used to explain how the selected design would work in practice. Although the focus here is on a particular surgical intervention and a specific trial, we believe that the methodology described will have wider application for many other clinical procedures in areas outside of the chosen setting.

### Clinical context

The rotator cuff is a group of muscles around the shoulder that help to stabilise the joint and initiate movement. Tears of the tendons of the rotator cuff, typically where they attach onto the humerus, are very common. Patients may present with persisting pain, loss of movement and substantial limitations in their activities of daily living. Treatment often consists of physiotherapy, but if this is not successful then surgery to repair the tear may be required. Sometimes the tears cannot be repaired, and there are very few effective treatments in this situation. Arthroscopic debridement has traditionally been used in this setting; it is an operation to clear space around the tendons and shoulder to allow it to move more freely and with less pain. There are concerns that this operation has little benefit over non-operative care [12], leading to calls for innovative solutions to treat this painful and disabling condition [13]. A newly available treatment option is a saline-filled balloon inserted above the main joint of the shoulder at the end of an arthroscopic debridement: the *InSpace* balloon device [14]. It is simple to deploy and adds less than 10 min to the operation. However, it is costly, and evidence for efficacy is scant [15]. It provides a cushion inside the shoulder joint that should improve biomechanics and hence reduce pain and improve shoulder function. We are running an adaptive, patient-assessor-blinded RCT across multiple centres in the UK, comparing standard arthroscopic debridement to standard arthroscopic debridement *plus* insertion of the *InSpace* balloon.

## Methods

### START:REACTS study

The START:REACTS study [16] (Sub-acromial spacer for Tears Affecting Rotator cuff Tendons: a Randomised, Efficient, Adaptive Clinical Trial in Surgery) commenced recruitment in autumn 2018; ISRCTN registration ISRCTN17825590 [17]. Recruitment is expected to take 24 months. In the following sub-sections we discuss important issues that motivated and determined the final study design, and provide a mathematical description of the methods that will be used to allow the possibility of early stopping.

#### Study outcomes

The primary outcome for the START:REACTS study is the Constant-Murley (C-M) shoulder score at 12 months [18, 19], which is widely used in trials, accepted by surgeons and has good reliability and responsiveness [20–23]; early outcomes will also be collected at 3 months and 6 months post-operation. Based on a recent meta-analysis, it is expected that the C-M score reaches a plateau by 12 months after intervention for a rotator cuff tear [24]. The scoring system consists of four sub-scales (pain, activities of daily living, strength and range of motion) that are combined to give a score out of 100 (perfect function).

#### Sample size

A minimum clinically important difference (MCID) for the Constant-Murley (C-M) score of 10 units has been widely used for other trials [12, 25, 26]. For purposes of analysis, the C-M score is considered to be approximately normally distributed with a standard deviation of 20, giving a moderate standardised mean difference of 0.5 [12, 27]. A recent meta-analysis [24] reported that standard deviations did not differ much between 3, 6 and 12 months, which is consistent with our own more detailed analysis of data available from another study reporting C-M scores [26]. For a costly invasive procedure of this nature, an effect size smaller than 10 units is unlikely to be considered worthwhile. For a power of 90% to detect an effect of this size and a two-sided type I error rate of 5%, a study without early stopping would require 170 participants (85 in each intervention group). The START:REACTS study was initially powered on this basis, with a 20% allowance for some loss to follow-up, giving a maximum sample size of 212.

Recruitment is planned to take 24 months at 15 centres; recruitment will begin with a single centre at month 1, increasing to 2 centres at 2 months, 3 centres at 3 months, 6 centres at 4 months, 9 centres at 5 months, 12 centres at 6 months and 15 centres at 7 to 24 months. There will be a total of 303 months of recruitment, which, assuming a constant recruitment rate at each centre, for a target of 170 participants means a rate of (approximately) 0.56 participants per centre per month.

Pilot work from a survey of shoulder surgeons, undertaken immediately prior to the start of the study, indicated that a treatment difference in the range 7.5–10 points on the C-M scale provided moderate to strong evidence in favour of the balloon intervention. Therefore, when considering options for stopping boundaries for the adaptive design, we would want to set these boundaries such that we had a low probability of stopping for futility for effect sizes of this magnitude, whilst at the same time stopping with high probability (for futility) for treatment differences in the range 0–2.5 points on the C-M scale.

#### Correlations between early and long-term outcomes

The best available evidence for correlations between early endpoints and the variance of the C-M shoulder score at 3, 6 and 12 months comes from a study undertaken in an analogous setting but in a different population to that planned for the START:REACTS study [26]. These data give estimates for the correlation between C-M shoulder scores at 3 and 6 months as *ρ*_3*m*,6*m*_=0.51, between 6-month and 12-month scores as *ρ*_6*m*,12*m*_=0.59 and between 3-month and 12-month scores as *ρ*_3*m*,12*m*_=0.46. Therefore, for the purposes of the simulations exploring the characteristics of the adaptive designs, we will assume a uniform correlation model (i.e. correlations between 3-, 6- and 12-month data are equal) with a value of 0.5.

#### Stopping window

The likely pattern of recruitment suggests that the window of opportunity for early stopping for the START:REACTS study will be relatively short. Presuming collection of primary 12-month outcome data commences promptly and proceeds to plan, and as we will not want to take an interim look before some 12-month data are available, it is likely that only after 18 months of recruitment could early looks at the data begin. Early looks at the data will need to complete by the end of recruitment at 24 months. Therefore, in practice, there will likely be a period of approximately 6 months when early looks at the data are possible. If this is the case, then the feasible number of early looks at the data will be small. Therefore, for the simulations exploring the characteristics of the adaptive designs, we will assume that there are either one, two or three early looks at the data.

### Statistical model

In the START:REACTS study the early endpoints at 3 and 6 months are monitored in addition to the primary 12-month endpoint. At the time of an interim analysis, before recruitment is complete, many more participants will have early endpoint data than 12-month (primary) endpoint data. Although the 3- and 6-month early endpoint data are useful for monitoring purposes, participant retention and safety issues, from a clinical perspective a treatment effect observed at 3 or 6 months will not necessarily translate to a treatment effect at the definitive 12-month endpoint; i.e. early benefit for the active intervention may not be sustained to the primary (clinically relevant) 12-month endpoint. Therefore, at the early looks we wish to gain information on the final 12-month endpoint from the early endpoints based on their expected within-participant correlations, irrespective of any early treatment effects. Stallard [9] shows that using early endpoint data, in a treatment selection (phase II/III) setting, leads to increases in power when these data are correlated with the primary endpoint, even if treatment effects on endpoints are unrelated. In the following sections we briefly outline the methods developed by Stallard [9] to control the familywise error rate in this setting and provide explicit expressions to estimate test statistics when there are two early endpoints.

#### Distribution of outcomes

Suppose participants in a study are followed up and data are collected on the same endpoint at a number of occasions; then let *X*_*ijK*_ be the final long-term outcome and *X*_*ij*1_…*X*_*ij*(*K*−1)_ be *K*−1 early (short-term) outcomes for participant *i* in intervention arm *j*. We assume outcomes are independent for different participants and that the distribution of outcomes (*X*_*ij*1_,⋯,*X*_*ijK*_) is multivariate normal, with mean (*μ*_1*j*_,⋯,*μ*_*Kj*_) and variance

$$\begin{array}{*{20}l}\left(\begin{array}{cccc} \sigma^{2}_{1} & \sigma_{1} \sigma_{2} \rho_{12} & \cdots & \sigma_{1} \sigma_{K} \rho_{1K}\\ \sigma_{2} \sigma_{1} \rho_{21} & \sigma^{2}_{2} & \cdots & \sigma_{2} \sigma_{K} \rho_{2K} \\ \vdots & \vdots & \ddots & \vdots \\ \sigma_{K} \sigma_{1} \rho_{K1} & \sigma_{K} \sigma_{2} \rho_{K2} & \cdots & \sigma^{2}_{K} \end{array}\right), \end{array} $$

where $\sigma ^{2}_{k}$ is the variance of the outcome *X*_*k*_ and $\rho _{kk^{\prime }}$ is the correlation between endpoints *X*_*k*_ and $X_{k^{\prime }}$.

#### Test statistic

For a two-arm study, participants are randomised to either the control (*j*=0) or active intervention (*j*=1) arms, and at an interim analysis, long-term (final) outcomes are available from *N*_*K*_ subjects and early (short-term) outcomes from *N*_1_…*N*_*K*−1_ subjects in each arm of the study. For our settings of interest, we assume that, at any time during follow-up, *N*_1_≥*N*_2_≥⋯≥*N*_*K*_; i.e. there are always more or equal numbers of subjects providing data for the earlier outcome *X*_*k*−1_ than the later outcome *X*_*k*_. The parameter of primary interest is the effect of the test intervention on the long-term (primary) outcome *X*_*K*_. Following Galbraith and Marschner [11], the treatment effect *B*, which uses all the available early endpoint data for two short-term outcomes (*X*_1_ and *X*_2_), for instance at 3 and 6 months such as in our chosen setting, and a single long-term outcome *X*_3_ (at 12 months) is given by:

1 $$ {\begin{aligned} B &= \frac{1}{N_{3}} \left[ \sum\limits_{i=1}^{N_{3}} (X_{i13} - X_{i03}) \qquad \qquad \qquad \qquad \qquad \qquad \right.\\ &\quad+\rho_{13} \frac{\sigma_{3}}{\sigma_{1}} \sum\limits_{i=N_{3}+1}^{N_{1}} \left(X_{i11} - X_{i01} - \frac{1}{N_{1}} \sum\limits_{m=1}^{N_{1}} (X_{m11} - X_{m01}) \right) \\ &\quad+\left.\rho_{23} \frac{\sigma_{3}}{\sigma_{2}} \sum\limits_{i=N_{3}+1}^{N_{2}} \left(X_{i12} \,-\, X_{i02} \,-\, \frac{1}{N_{2}} \sum\limits_{m=1}^{N_{2}} (X_{m12} - X_{m02}) \right) \right], \end{aligned}}  $$

with variance:

2 $$ {\begin{aligned} \text{var}(B) = \frac{2 \sigma^{2}_{3}}{N_{3}} \left[ 1 - \rho^{2}_{13} \frac{N_{1} - N_{3}}{N_{1}} - \rho^{2}_{23} \frac{N_{2} - N_{3}}{N_{2}} + \right.\\ \left.2 \rho_{13} \rho_{23} \rho_{12} \left(1 - \frac{N_{3}}{N_{2}}\right) \right]. \end{aligned}}  $$

Estimates $\hat {B}$ and $\text {var}(\hat {B})$ follow from estimates of the correlations *ρ*_13_, *ρ*_23_ and *ρ*_12_ and standard deviations, *σ*_1_, *σ*_2_ and *σ*_3_, obtained from the appropriate regression models, using all available data. Expressions (1) and (2) are presented for the special case of equal numbers of subjects in each arm of the study. However, they can be modified easily for the case of unequal numbers in the study arms. These and more general expressions for *B* and var(*B*) for *K*−1 early outcomes are provided in Additional file 1. From expressions (1) and (2) it is clear that if long-term outcome *X*_3_ is uncorrelated with short-term outcomes *X*_1_ and *X*_2_ (i.e. if *ρ*_13_=*ρ*_23_=0), then *B* and var(*B*) simplify to conventional expressions we would use to estimate the mean treatment effect (and variance) for *X*_3_ alone, without reference to the early endpoints. As correlations between *X*_3_ and *X*_1_ and *X*_2_ increase in magnitude, then var(*B*) decreases, provided that the two early outcomes *X*_1_ and *X*_2_ are not themselves strongly correlated. In general, var(*B*) is minimised as both *ρ*_13_→1 and *ρ*_23_→1, and *ρ*_12_→0; i.e. *X*_1_ and *X*_2_ are strongly correlated with *X*_3_, but are themselves uncorrelated.

### Implementation for a two-arm trial

For a two-arm study, with two short-term outcomes, study participants are randomised to either the control or active intervention arms. Data collection proceeds until the first interim analysis when *N*_31_ long-term data and *N*_11_ and *N*_21_ short-term data are available per arm; *N*_3*w*_, *N*_2*w*_ and *N*_1*w*_ are the number of study participants with long and short-term data available at early look *w*. Expressions (1) and (2) are used to obtain the test statistic $S_{1} = \hat {B}_{1}/\text {sd}\left (\hat {B}_{1}\right)$ and observed information $\hat {I}_{1} = 1/{\text {var}\left (\hat {B}_{1}\right)}$, using estimates $\hat {\sigma }^{2}_{3}$, $\hat {\rho }_{12}$, $\hat {\rho }_{13}$ and $\hat {\rho }_{23}$ obtained from the observed data. The observed test statistic is then compared to pre-defined lower and upper stopping boundaries *l*_1_ and *u*_1_, which are determined by the expected information *I*_1_ at the first look, and either the trial is stopped, for futility or efficacy, or it continues to the next interim analysis. At each subsequent interim analysis, the test statistic $S_{w} = \hat {B}_{w}/\text {sd}\left (\hat {B}_{w}\right)$ is calculated in the same way as in the first analysis, using all available data on short-term and long-term outcomes, and compared to stopping boundaries *u*_*w*_ and *l*_*w*_ that determine whether the study is stopped early. If the trial is stopped early at an interim analysis, then long-term data will continue to be collected on all those recruited up to that point, and these data will be used for final (definitive) inferences in an overrunning analysis [28].

The timing of the first and subsequent looks is typically specified at the commencement of the study via the selected values for *N*_3*w*_, *N*_2*w*_ and *N*_1*w*_ at each early look *w*. These values are used, together with expected values of $\sigma ^{2}_{3}$, *ρ*_12_, *ρ*_13_ and *ρ*_23_, to give the expected information *I*_*w*_ at each planned early look *w*, using expression (2). The observed information $\hat {I} = 1/{\text {var}\left (\hat {B}\right)}$ is monitored during data accrual, and interim analysis *w* occurs when the observed information equals the expected information at look *w* (see later Worked example).

### Sequential stopping boundaries

We are interested in a sequential trial with two short-term endpoints where a series of *W* interim analyses (looks) are undertaken to compare the two groups. The number of study participants increases in the two groups, and thus the long-term and short-term data available for analysis also increase through the course of the trial. Tests are performed at each of a series of interim analyses in order to make inferences about the superiority of the active intervention group (over the control) in terms of the long-term endpoint. The tests are undertaken at interim analysis *w*, using test statistic *S*_*w*_, and must control the type I error rate across the *W* interim analyses. For a one-sided alternative at overall level *α*, with possible stopping for futility, the type I error rate spent is such that $\alpha ^{*}_{U}(1) < \cdots < \alpha ^{*}_{U}(W) = \alpha $ and $\alpha ^{*}_{L}(1) < \cdots < \alpha ^{*}_{L}(W) = 1 - \alpha $, where $\alpha ^{*}_{U}(w)$ is the probability of stopping and rejecting *H*_0_ in favour of *B*>0 at look *w* (efficacy), and $\alpha ^{*}_{L}(w)$ is the probability of stopping without rejecting *H*_0_ at look *w* (futility). The type I error rates spent are determined by $\alpha ^{*}_{U}(w)$ and $\alpha ^{*}_{L}(w)$, which are specified in advance of the study beginning. Stallard [9] proposes a method for construction of stopping boundaries in this scenario for the more general setting of *T* intervention arms and a single control arm. For a two-arm study, standard group sequential methods and widely available software allow one to calculate the lower and upper stopping boundaries (*l*_*w*_ and *u*_*w*_) at each look *w* [29].

### Simulations

The statistical methodology described here provides a framework for how decisions about early stopping will be made. In order to understand how our assumptions about the likely size of the treatment effect, settings for nuisance parameters and the number of planned interim analyses will affect design characteristics (e.g. how often we stop early for futility), we simulate data from the full multivariate distribution of outcomes (*X*_*ij*1_,⋯,*X*_*ijK*_) for each of the *i* study participants and undertake interim and final analyses many times. A Poisson model [30] is used to simulate the likely pattern of participant recruitment into the study. A constant monthly recruitment rate at each centre is assumed, with a smooth increase up to the target number of centres during the first 6 months of the planned 24 months of recruitment. The pattern of follow-up data collection at 3, 6 and 12 months is assumed to mirror that for recruitment. The timing of the interim looks are set at the start of a study using selected (feasible) values for *N*_3_ and, based on the expected patterns of early data accrual, *N*_2_ and *N*_1_. These together with the expected values of *ρ*_12_, *ρ*_13_, *ρ*_23_ and *σ*_3_ determine the expected information content of the data at each look *I*_*w*_=1/var(*B*_*w*_), using expression (2). The pre-specified stopping boundaries follow directly from *I*_*w*_, $\alpha ^{*}_{L}$ and $\alpha ^{*}_{U}$. The temporal pattern of participant recruitment, data collection and ultimately information are simulated for a single realisation of the study. For each simulation, a series of estimates for *ρ*_12_, *ρ*_13_, *ρ*_23_ and *σ*_3_ are calculated using progressively increasing amounts of data as each new participant is recruited into the study. The pattern of (simulated) information accrual follows from these estimates and the temporal pattern of data collection, using expression (2).

Interim looks at the data occur when the simulated information is equal to the information content at the pre-specified stopping boundaries. The estimated test statistics are compared to stopping boundaries, with decisions on stopping following directly from these comparisons. Thus, the simulations emulate how the study would have evolved, and how decisions about stopping would have been made in a manner as close to a real-life setting as we can feasibly create. Undertaking these simulated analyses many times allows us to estimate expected stopping probabilities and overall power (to reject the null hypothesis) that inform our decisions about the overall study design.

## Results

### Recruitment and data accrual

**Fig. 1:**
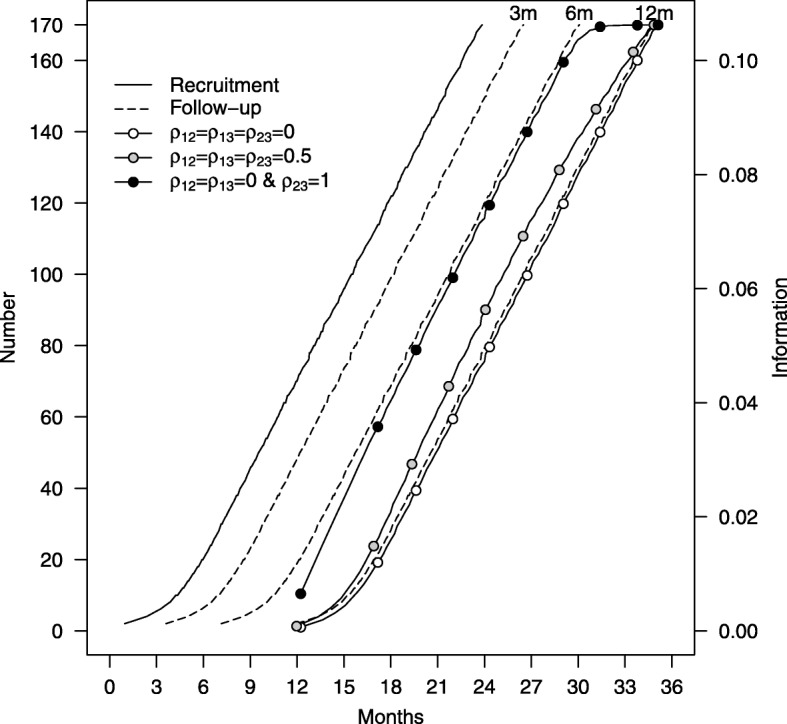
Recruitment, data and information accrual during follow-up. Expected recruitment, data and information accrual during 24 months, estimated from simulations. Information accrual is plotted for three possible correlation models: *ρ*_12_=*ρ*_13_=*ρ*_23_=0.5, *ρ*_12_=*ρ*_13_=*ρ*_23_=0 and, *ρ*_12_=*ρ*_13_=0 and *ρ*_23_=1

Simulating data from the recruitment model suggested that within the window of opportunity for early stopping (between 18 and 24 months from commencement of recruitment), 12-month data will be available from between 15 and 40 participants per intervention arm (*N*_3_). Figure 1 shows the expected patterns of recruitment, data and information accrual during follow-up for our chosen correlation model *ρ*_12_=*ρ*_13_=*ρ*_23_=0.5, obtained from the simulations. The figure also shows information accrual (i.e. 1/var(*B*)) for two extreme scenarios, where (1) *ρ*_12_=*ρ*_13_=*ρ*_23_=0 and (2) *ρ*_12_=*ρ*_13_=0 and *ρ*_23_=1, that represent the patterns of accrual when the early outcomes (3 months and 6 months) provide no information on the final 12-month outcome and when the 6-month outcome is exactly the same as the 12-month outcome. In these two scenarios the patterns of information accrual are for scenario (1) exactly as would be observed if the 12-month outcome only provided all the relevant information, and in scenario (2) exactly as would be observed if all the information were provided by the 6-month data alone. For purposes of motivating the simulations, it is useful to divide the likely recruitment numbers available in the window of opportunity for early stopping interval (a period of 6 months) equally. Figure 1 indicates the likely patterns of data accrual at six potential interim looks for 12-, 6- and 3-month data to be approximately as follows: at the first possible look *N*_3_=15, *N*_2_=35 and *N*_1_=50, at the second look *N*_3_=20, *N*_2_=40 and *N*_1_=55, at the third look *N*_3_=25, *N*_2_=45 and *N*_1_=60, at the fourth look *N*_3_=30, *N*_2_=50 and *N*_1_=65, at the fifth look *N*_3_=35, *N*_2_=55 and *N*_1_=70 and at the sixth look *N*_3_=40, *N*_2_=60 and *N*_1_=75. Under the expected correlation model *ρ*_12_=*ρ*_13_=*ρ*_23_=0.5 and expected standard deviation of the 12-month outcome (*σ*_1_=20), the information at each of these possible looks at the data is 21.4%, 28.0%, 34.4%, 40.8%, 47.1% and 53.3%, expressed as a percentage of the expected information at the study endpoint given by ${N}/{2 \sigma ^{2}_{3}}= 85/800=0.106$. If *ρ*_12_=*ρ*_13_=*ρ*_23_=0, then this reduces to 17.6%, 23.5%, 29.4%, 35.3%, 41.2% and 47.1%; a correlation of 0 implies there is no information, on 12-month outcomes, from the early 3- and 6-month outcomes.

### Type I error rate

As a prelude to simulations exploring overall study power and as a check of the software implementation, a number of simulations were undertaken to explore study characteristics under the null hypothesis (no treatment effect). The results of these simulations, for a selection of three likely data accrual patterns, are shown in Table 1. It is apparent from Table 1 that the estimated type I error rates for the three selected settings (1) one early look *N*_1_=60,*N*_2_=45,*N*_3_=25, (2) two early looks *N*_1_=(55,70),*N*_2_=(40,55),*N*_3_=(20,35) and (3) three early looks *N*_1_=(50,65,75),*N*_2_=(35,50,60), *N*_3_=(15,30,40) are well controlled at the 2.5% level. Also, the estimated cumulative probabilities of stopping for futility at early looks *p*_*w*,*F*_ are equal (within simulation error) to the pre-specified lower error spending values, $\alpha ^{*}_{L}$.

**Table 1:** Estimated type I error rates, where *p*_*w*,*F*_ is the cumulative probability of stopping for futility at look *w* or earlier, *p*_*E*_ is the probability of stopping early for efficacy and *p*_12*m*_ is the probability of stopping for efficacy at the end of the study; *N*=85, for (a) one look *N*_1_=60,*N*_2_=45,*N*_3_=25, (b) two looks *N*_1_=(55,70),*N*_2_=(40,55),*N*_3_=(20,35) and (c) three looks, *N*_1_=(50,65,75),*N*_2_=(35,50,60),*N*_3_=(15,30,40), *ρ*=*ρ*_13_=*ρ*_23_=*ρ*_12_ and $\sigma ^{2}_{1} = \sigma ^{2}_{2} = \sigma ^{2}_{3} = 20$ (10,000 simulations)

Futility bound ($\alpha ^{*}_{L}$)	*ρ*	*p*_*E*_	*p*_1,*F*_	*p*_2,*F*_	*p*_3,*F*_	*p*_12*m*_
(a) One look; $\alpha ^{*}_{U}=(0.001,0.025)$						
(0.0,0.975)	0.0	0.002	0.000	-	-	0.025
(0.5,0.975)	0.0	0.002	0.504	-	-	0.023
(0.0,0.975)	0.5	0.002	0.000	-	-	0.026
(0.5,0.975)	0.5	0.002	0.504	-	-	0.026
(b) Two looks; $\alpha ^{*}_{U}=(0,0.001,0.025)$						
(0.0,0.0,0.975)	0.0	0.001	0.000	0.000	-	0.025
(0.2,0.5,0.975)	0.0	0.001	0.202	0.499	-	0.025
(0.0,0.0,0.975)	0.5	0.001	0.000	0.000	-	0.024
(0.2,0.5,0.975)	0.5	0.002	0.199	0.505	-	0.025
(c) Three looks; $\alpha ^{*}_{U}=(0,0,0.001,0.025)$						
(0.0,0.0,0.0,0.975)	0.0	0.001	0.000	0.000	0.000	0.024
(0.1,0.3,0.5,0.975)	0.0	0.002	0.110	0.306	0.503	0.025
(0.0,0.0,0.0,0.975)	0.5	0.001	0.000	0.000	0.000	0.025
(0.1,0.3,0.5,0.975)	0.5	0.001	0.108	0.307	0.506	0.025

### Power

**Fig. 2:**
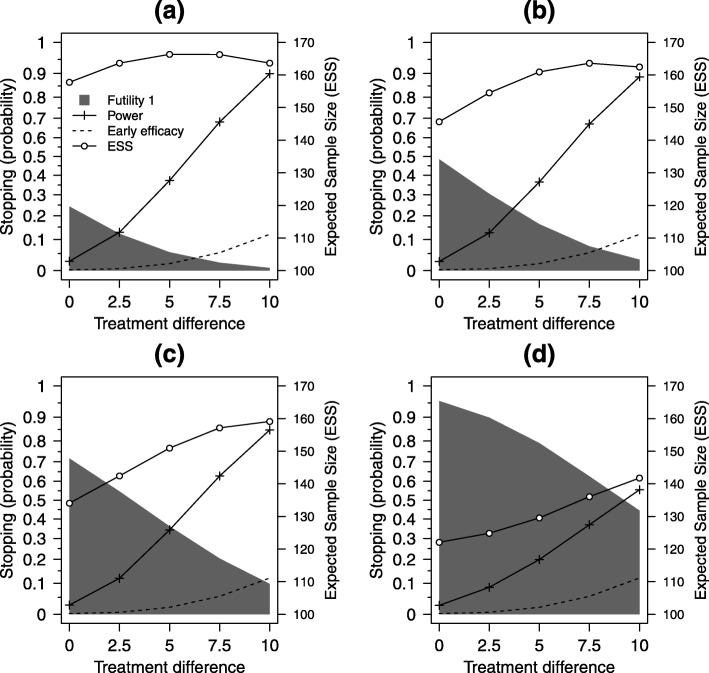
Design characteristics for one early look. Estimated probabilities of stopping for *futility* and *efficacy* at the first look, expected sample size (ESS) and overall study power, for effect sizes in range 0 to 10 for **a**$\alpha ^{*}_{L}=(0.24,0.975)$, **b**$\alpha ^{*}_{L}=(0.48,0.975)$, **c**$\alpha ^{*}_{L}=(0.72,0.975)$ and **d**$\alpha ^{*}_{L}=(0.96,0.975)$. Here $\alpha ^{*}_{U}(1)=0.001$, *ρ*=0.5; other settings are as in Table 1

**Fig. 3:**
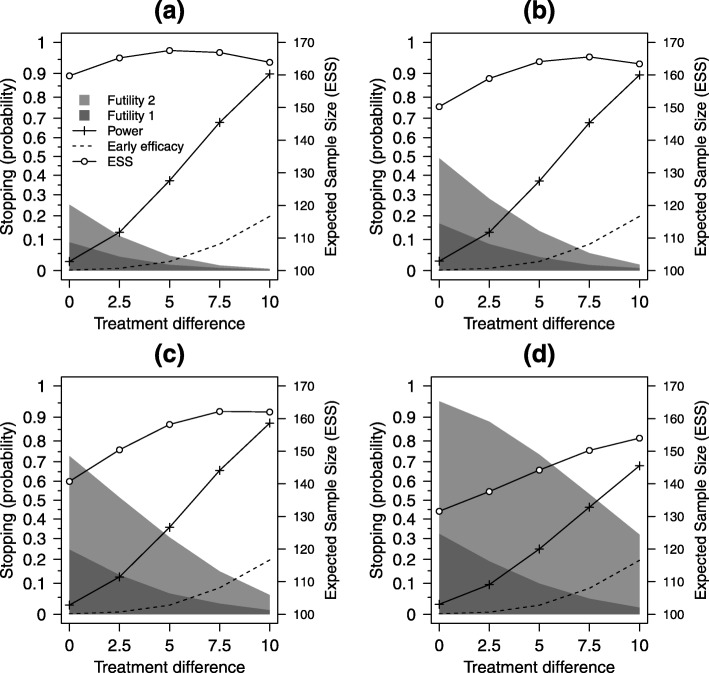
Design characteristics for two early looks. Estimated probabilities of stopping for *futility* and *efficacy* at the first and second looks, expected sample size (ESS) and overall study power, for effect sizes in range 0 to 10 for **a**$\alpha ^{*}_{L}=(0.08,0.24,0.975)$, **b**$\alpha ^{*}_{L}=(0.16,0.48,0.975)$, **c**$\alpha ^{*}_{L}=(0.24,0.72,0.975)$ and **d**$\alpha ^{*}_{L}=(0.32,0.96,0.975)$. Here $\alpha ^{*}_{U}(1)=0$ and $\alpha ^{*}_{U}(2)=0.001$, *ρ*=0.5; other settings are as in Table 1

**Fig. 4:**
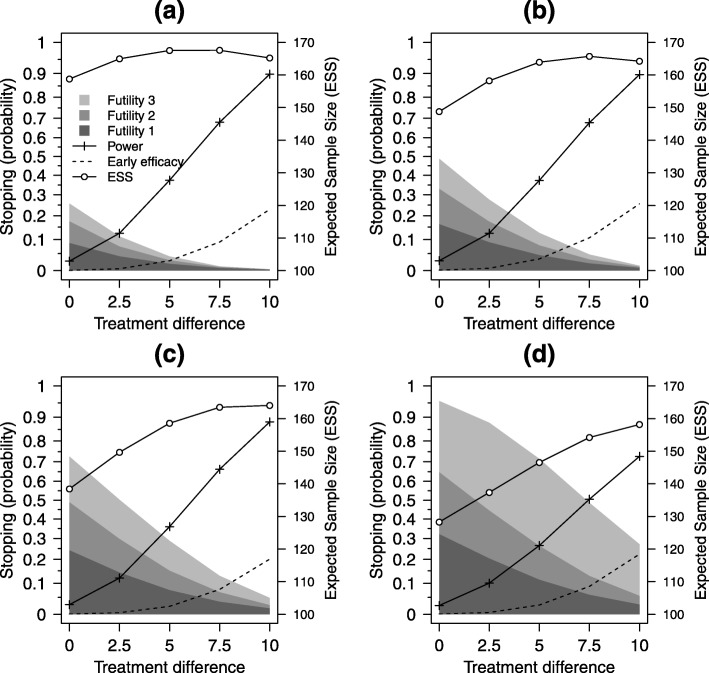
Design characteristics for three early looks. Estimated probabilities of stopping for *futility* and *efficacy* at the first, second and third looks, expected sample size (ESS) and overall study power, for effect sizes in range 0 to 10 for **a**$\alpha ^{*}_{L}=(0.08,0.16,0.24,0.975)$, **b**$\alpha ^{*}_{L}=(0.16,0.32,0.48,0.975)$, **c**$\alpha ^{*}_{L}=(0.24,0.48,0.72,0.975)$ and **d**$\alpha ^{*}_{L}=(0.32,0.64,0.96,0.975)$. Here $\alpha ^{*}_{U}(1)=0$, $\alpha ^{*}_{U}(2)=0$ and $\alpha ^{*}_{U}(3)=0.001$, *ρ*=0.5; other settings are as in Table 1

Overall study power and stopping probabilities were estimated for a range of plausible 12-month treatment differences for the C-M score scale (0, 2.5, 5, 7.5 and 10); these corresponded to standardised effect sizes, for the selected value of *σ*_*Y*_=20, of 0, 0.125, 0.25, 0.375 and 0.5. A range of values for the lower bounds $\alpha ^{*}_{L}$ were tested for one, two and three early looks at the data, using the same values for *N*, *N*_3_, *N*_1_ and *N*_2_ as described previously for type I error rate estimation, using the uniform correlation model (*ρ*=*ρ*_13_=*ρ*_23_=*ρ*_12_) with a value of *ρ*=0.5. Efficacy stopping boundaries were set to $\alpha ^{*}_{U}=(0.001,0.025)$, $\alpha ^{*}_{U}=(0,0.001,0.025)$ and $\alpha ^{*}_{U}=(0,0,0.001,0.025)$, at one, two and three early looks respectively. The main initial clinical focus of our design is to determine whether the balloon procedure is ineffective or harmful. Therefore, the emphasis in the simulations and in the planned designs will be on early stopping for futility, which is determined by $\alpha ^{*}_{L}$. The chosen settings for the upper (efficacy) boundaries $\alpha ^{*}_{U}$ favour collecting as much information as possible if there is emerging evidence of efficacy. Early stopping for efficacy will only be considered at the last interim look, with boundaries set such that only if there is very strong evidence that the balloon procedure is superior to standard care will early stopping be considered. Figure 2 shows results for one early look at the data, Fig. 3 for two early looks at the data and Fig. 4 for three early looks at the data.

There are strong trends for increasing power as the treatment difference increases from 0 to 10 points on the C-M score scale and corresponding decreases in the futility stopping probabilities. Estimates for early stopping for efficacy from the simulations, which were planned for the last of the interim looks only, increased from approximately 10% for one early look to 20% for two early looks and 25% for three early looks, for a treatment difference of 10 points. This was due to more data being available at the look when stopping for efficacy can occur (*n*=15 for one look, *n*=35 for two looks and *n*=40 for three looks).

Four options for futility stopping were investigated for $\alpha ^{*}_{L}$ that represented a sequence of increasingly aggressive options, from a low probability of stopping, labelled as (a), to a high probability, labelled as (d), with (b) and (c) intermediate to these. For one early look at the data, $\alpha ^{*}_{L}$ was set to either (a) (0.24,0.975), (b) (0.48,0.975), (c) (0.72,0.975) or (d) (0.96,0.975), for two early looks to either (a) (0.08,0.24,0.975), (b) (0.16,0.48,0.975), (c) (0.24,0.72,0.975) or (d) (0.32,0.96,0.975) and for three early looks to either (a) (0.08,0.16,0.24,0.975), (b) (0.16,0.32,0.48,0.975), (c) (0.24,0.48,0.72,0.975) or (d) (0.32,0.64,0.96,0.975).

Under the null hypothesis (C-M treatment difference equal to 0), $\alpha ^{*}_{L}$ represented the expected stopping probabilities (for futility) at each look. For the largest treatment differences (10 on C-M score scale) and the most aggressive stopping options, the futility stopping rates were 44.4% for one early look (Fig. 2d), 31.9% for two early looks (Fig. 3d) and 27.1% for three early looks (Fig. 4d). For this most aggressive futility stopping setting, study power was lowered significantly due to (incorrect) early stopping. Power was reduced to only 55.5%, 68.0% and 72.7%, in these three settings, rather than the 90% we would expect for a non-adaptive design. The least aggressive futility stopping option (Figs. 2a, 3a and 4a) showed good power (89.5%, 89.7% and 89.7%) but poor early stopping under the null hypothesis (24.3%, 25.1% and 26.7%). The two extreme futility stopping options (Figs. 2a, d 3a, d and 4a, d), therefore, do not have the characteristics we are seeking in the design.

The intermediate options (Figs. 2b, c 3b, c and 4b, c), however, have more desirable characteristics, as they have reasonable power for a strong treatment effect (C-M treatment difference of 10) whilst retaining the ability to stop early for futility, with high probability, under the null hypothesis. For example, for two early looks when $\alpha ^{*}_{L}=(0.24,0.72,0.975)$ (Fig. 3c), overall power was 87.6% for a treatment difference of 10, with a stopping rate of 24.5% at the first look and 72.9% at the first or second look combined.

The expected sample size (ESS), calculated from the expected stopping probabilities and expected pattern of patient and data accrual, provides a useful summary of the design characteristics that complements study power. The right-hand *y*-axes of Figs. 2, 3 and 4 are annotated to provide a useful informal comparator to the fixed study design with a sample size of 170; this provides 90% power to detect a C-M score treatment difference of 10 points between intervention arms, at the 5% level. The ESS decreases, for all numbers of early looks, from the least (a) to the most aggressive (d) futility stopping options; increasing the probability of stopping early, for either futility or efficacy, lowers the overall study sample size from that we would need for the non-adaptive (fixed) study design (sample size 2*N*=170). The pattern of variation for ESS, across treatment differences, reflects the dominance of either futility stopping (for zero and small differences) or efficacy (for large differences). In selecting a good design, we aim to find settings of the stopping boundaries that maintain overall power at or as close as possible to the nominal (non-adaptive) 90% level, whilst at the same time lowering the expected sample size across the range of treatment effects we might expect to see in the study.

The number of study participants required to reach the required information levels at the early looks was also assessed in the simulations. The expected (mean) numbers were very close to the sample sizes used to motivate the simulations, as we would expect, i.e. *N*_3_=25 for one early look, *N*_3_=(20,35) for two early looks and *N*_3_=(15,30,40) for three early looks. The simulations were set up such that early looks at the data took place even if recruitment had been completed, whereas in reality, the early looks would have been abandoned. Recruitment had been completed at the final early look at the data for (approximately) 0%, 3% and 12% of the simulations for one, two and three early looks. The high value for three early looks reflects the fact that the final early look at the data occurs when approximately 40 participants in each arm of the study have 12-month outcome data, which is quite close to 50, the point when the recruitment model expects that recruitment will have completed.

### Worked example

In order to illustrate how the design will work in practice, we briefly work through the necessary calculations, using purely synthetic data, for a much smaller and simpler example than those used in the simulations. The data and R code [31] for implementation are provided in Additional file 1.

A study is planned with $\alpha ^{*}_{L}=(0.200,0.600,0.975)$ and $\alpha ^{*}_{U}=(0.000,0.001,0.025)$ for two early looks, with group sample sizes of *N*_3_=(10,15), *N*_2_=(15,20), *N*_1_=(20,25) and *N*=30; we assume equal group sizes, and two early outcomes and a final outcome as previously, for ease of exposition. Let us suppose that data available from a pilot study suggest correlations between outcomes of *ρ*_13_=*ρ*_23_=0.5 and *ρ*_12_=0, with *σ*_3_=18. Using these values in expression (2) indicates that the expected information at the early looks will be *I*_1_=0.019 and *I*_1_=0.028, and at the final analysis $\textit {I}_{\text {Final}} = {N}/{2 \sigma ^{2}_{Y}}= 30/648=0.046$ (for *σ*_*Y*_=18). Expressed as a percentage of the information available at the final analysis, this corresponds to 42% and 60%, for the two early looks. The boundaries can be calculated using widely available software, for instance the gsDesign [32] package in R. For our selected values for $\alpha ^{*}_{L}$ and $\alpha ^{*}_{U}$ and the expected information at our planned looks, the function gsBound provides the following boundaries for decision making: at look 1, *l*_1_=−0.842 (lower boundary) and *u*_1_=*∞* (upper boundary), at look 2 *l*_2_=0.247 and *u*_2_=3.09, and at the final analysis *l*_Final_=*u*_Final_=1.96.

**Fig. 5:**
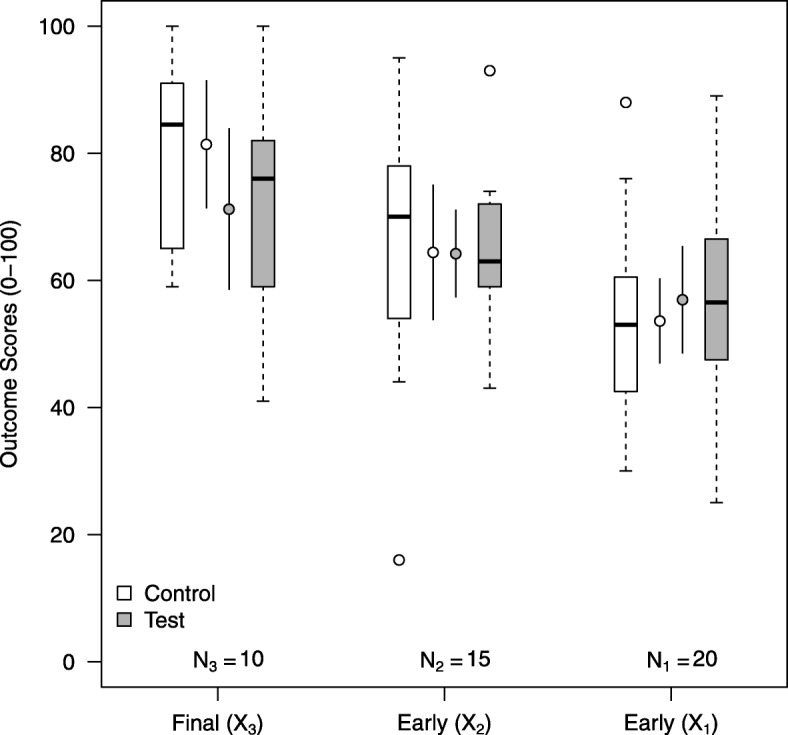
Outcome score data at the first look. Boxplots and means with 95% confidence intervals of early (*X*_1_ and *X*_2_) and final (*X*_3_) outcome data by intervention group at the first interim analysis

Data collection proceeded as planned, with information monitored during follow-up. After the twentieth participant had provided final outcome data, the estimated information (0.02) reached the pre-set value for the first look (0.019). Figure 5 shows the distributions of outcome data at the first look. The estimate of the mean treatment difference (in favour of the test group) for the final outcome (*X*_3_) was –10.2; i.e. the outcome score for the test intervention was considerably lower than that for the control intervention. Estimates of the correlations between outcomes and the standard deviation of the final outcome were as follows: $\hat {\rho _{13}} = 0.45$, $\hat {\rho _{23}} = 0.20$, $\hat {\rho _{12}} = 0.04$ and $\hat {\sigma }_{3}=16.8$. Calculating *B* and var(*B*), using expressions (1) and (2)), provides estimates of the mean treatment difference for the outcome of –9.77, with variance 50.18 (see Additional file 1). Therefore, the test statistic at look 1, *S*_1_=−1.38, is less than the lower boundary (–0.842), indicating that the study should be *stopped* for futility.

Continuing to follow up all those in the study, after the decision to stop at look 1, in an *overrunning* analysis [28] provides estimates of *B*=−3.70 and var(*B*)=20.5 (*p*=0.419). This confirms that the decision made to stop at look 1 appears to have been correct and leads us to conclude that there is no evidence that the test group performs better than the control group.

If different settings for $\alpha ^{*}_{L}$ had been selected, then the study may have proceeded in a different manner. For instance, if a less aggressive lower stopping criterion had been used at the first look (e.g. $\alpha ^{*}_{L}=(0.080,0.600,0.975)$), then the lower boundary at the first look would be *l*_1_=−1.41, and the study would not have stopped for futility.

## Discussion

This manuscript describes work to develop an adaptive clinical trial design motivated by a trial for testing a novel surgical approach for repair of rotator cuff tendon tears. The design, which builds and expands on previously published methodology [9, 11], uses early observations of the primary outcome at 3 months and 6 months to augment 12-month outcome data to inform decision making on early stopping. The main focus in the development of the design is on *futility* stopping, rather than *efficacy* stopping; i.e. stopping for *efficacy* in the simulations is limited to the last interim look at the data and is such that very strong evidence is required to stop. This reflects the clinical perspective that if a new intervention shows promise, then it is prudent, within reason, to continue to collect data to the planned study sample size, rather than stop early, in order to provide more precise effect estimates and increase the chances of detecting any adverse events.

The simulations showed that with more looks at the data the chance of recruitment completing before the final look increased; recruitment completed before the final look in 3% and 12% of simulations for two and three early looks. More looks offer more possibilities for early decision making, but at a greater risk of not completing the planned early looks before the end of recruitment. The estimated rates of recruitment completing before the last early look are clearly in part at least dependent on the veracity of the recruitment model. If recruitment was much higher or faster than expected at times during recruitment, then this could be problematic for the design. For instance, a rapid unexpected rise in the recruitment rate could cause recruitment to be completed before the early looks at the data had happened. We do not think this will happen in our setting, as there are structural (study-based) limitations in the number of centres, clinicians and timings of clinics which make this highly unlikely. However, recruitment will be monitored closely. In the START:REACTS study it is likely that early looks will be dropped if recruitment completes much more rapidly than expected. However, it may be desirable in other settings to close centres or temporarily suspend recruitment if this were feasible.

As with conventional sample size calculations, the results of the simulations are dependent on assumptions made about the variance of the primary outcome (12-month C-M score) and the correlations between the early 3- and 6- and 12-month scores. We have good evidence on these *nuisance* parameters from a recently published systematic review [24] and relevant data [26]. A larger than expected value has been deliberately selected for the 12-month C-M score standard deviation (*σ*_3_=20); close inspection of the data from [24] suggests that the standard deviation is likely to be nearer to 15 than 20. Conservatively, a value of 20 was chosen for the simulations. If *σ*_3_ is lower than 20, then we will reach the planned study information points, which determine the timings of the early looks at the data, sooner than the simulations indicate.

The simulations assume a relatively moderate correlation model for the study outcomes: *ρ*_13_=*ρ*_23_=*ρ*_12_=0.5. If the correlation model were stronger than expected (e.g. *ρ*_13_=*ρ*_23_=*ρ*_12_=0.9), and all other things were unchanged, we would reach the information thresholds for the early looks sooner than planned (i.e. with fewer participants) and potentially gain more from the adaptive design than we estimate from the simulations. Conversely, if the correlations are such that the early outcomes tell us nothing about the definitive outcome (i.e. *ρ*_13_=*ρ*_23_=*ρ*_12_=0), then we would accumulate information more slowly than the simulations suggest, and recruitment is likely to have completed before the information required for the first look at the data is reached. In such a setting the design would proceed to the fixed recruitment target in the conventional manner. The *loss* in such a setting would be the increase in sample size, relative to the fixed design, that we would need for the adaptive design, For example, for the START:REACTS study described previously, the sample size would need to increase from 170 participants to between 180 and 188, dependent on the choice of boundaries and early looks. This is a relatively modest increase in sample size for this study, given the potential gains from early stopping, but in other application areas this may be an unacceptable increase in sample size if there is little evidence for even moderate associations between the early and final study outcomes.

The simulations show that the error rate is controlled at the specified rate, provided that the stopping rules are *binding* [33]. Here, by *binding* we mean that stopping for futility at the early look is *essential* whenever the futility boundaries are crossed; irrespective, for instance, of reasons external to the study, such as new or emerging evidence on the interventions. The simulations show study power based on a sample size of 170 (85 in each group). This provided 90% power for the non-adaptive design. For the adaptive designs with appealing operating characteristics discussed here, the power is somewhat lower than 90%. For the definitive adaptive study design, the overall sample size will be increased to provide 90% power. The final selection of overall sample size, stopping boundaries and number of looks will be made by the START:REACTS data and safety monitoring committee (DSMC) and confirmed by the trial steering committee (TSC). The boundaries, timings of the interim looks and agreement on binding will be incorporated into the DSMC charter and will be kept confidential within the study team.

The work described here is focussed primarily on the design of the START:REACTS study, and this is reflected in the set-up of the simulations and data generating model. For instance, we have assumed that the correlations between the outcomes are the same within the intervention arms. This need not be the case in other applications, and it would be relatively straightforward to modify the set-up of the simulations to allow different correlations in the intervention arms or different variances for each of the early outcomes. We believe that the designs discussed will have much wider application in many analogous settings, particularly when trials are undertaken to assess new surgical and other interventions where outcomes are assessed over a long period of time. Typically in studies of this type designs are non-adaptive, and early outcomes, usually available as part of routine monitoring of patients, are simply reported as secondary outcomes. This is both inefficient and wasteful. With increased methodological understanding and availability and ease of use of software tools for implementing adaptive designs, we believe that this situation will change in the future.

## Conclusions

In this manuscript we present a methodology for the design of an adaptive clinical trial motivated by testing a novel surgical approach for repair of rotator cuff tendon tears. The design uses early observations of the 12-month primary outcome at 3 months and 6 months to augment 12-month outcome data to inform decision making on early stopping. We derive estimators for the treatment effect and test statistics based on the setting of two early outcomes, and present methods for estimation of sequential stopping boundaries. Simulations are undertaken for one, two and three early looks with a range of options for stopping boundaries. We show that a design with two early looks is feasible and, with appropriately chosen futility stopping boundaries, has appealing design characteristics. A number of possible design options are described that have good power and a high probability of stopping for futility if there is no evidence of a treatment effect at early looks. A worked example provides a practical demonstration of how the design might work in a real study. In summary, the work shows that an adaptive design is feasible and could work in practice, and it provides some guidelines for appropriate values for the stopping boundaries for the START:REACTS study.

## Supplementary information

**Additional file 1** General expressions for *B* and var(*B*) for *K*−1 early outcomes. Expressions *B* and var(*B*) for unequal group sizes for two early outcomes. R code for the worked example.

## Data Availability

The datasets used and analysed and the code written as part of this study are available from the corresponding author on reasonable request.

## Abbreviations

C-M: Constant-Murley shoulder score
DSMC: Data and safety monitoring committee
EME: (UK) Efficacy and Mechanism Evaluation (Programme)
ESS: Expected sample size
MCID: Minimum clinically important difference
MRC: (UK) Medical Research Council
NHMRC: (Australian) National Health and Medical Research Council
NICE: (UK) National Institute for Health and Care Excellence
NIHR: (UK) National Institute for Health Research
RCT: Randomised controlled trial
START: REACTS Sub-acromial spacer for Tears Affecting Rotator cuff Tendons: a Randomised, Efficient, Adaptive Clinical Trial in Surgery
TSC: Trial steering committee
