# Supplementary material for: An adaptive two-arm clinical trial using early endpoints to inform decision making: design for a study of sub-acromial spacers for repair of rotator cuff tendon tears
Source: Trials. 2019 Dec 9;20:694. doi: 10.1186/s13063-019-3708-6 (PMC6902495; doi:10.1186/s13063-019-3708-6)
Supplement: Supplementary file 1 — Additional file 1 General expressions for B and var(B) for K−1 early outcomes. Expressions B and var(B) for unequal group sizes for two early outcomes. R code for the worked example. [file 13063_2019_3708_MOESM1_ESM.pdf]

Additional File 1

Nick Parsons[1], Nigel Stallard[1], Helen Parsons[2], Philip Wells[2], Martin Underwood[2,3], James Mason[2] and Andrew Metcalfe[2,3]

B and var(B) for $K-1$ early outcomes

For a two-arm study, participants are randomized to either the control ($j = 0$) or active intervention ($j = 1$) arms, and at an interim analysis long-term (final) outcomes are available from N_K subjects and early (short-term) outcomes from $N_1 \ldots N_{K-1}$ subjects in each arm of the study. Assuming equal numbers of subjects providing data for the earlier outcome X_{k-1} than the later outcome X_K, the effect of the test intervention on the long-term (primary) outcome X_K [1] is given by

$$B = \frac{1}{N_K}\left[\sum_{i=1}^{N_K}(X_{i1K} - X_{i0K}) + \sum_{k=1}^{K-1}\left[\rho_{kK}\frac{\sigma_K}{\sigma_k} \sum_{i=N_K+1}^{N_k}\left(X_{i1k} - X_{i0k} - \frac{1}{N_k}\sum_{m=1}^{N_k}(X_{m1k} - X_{m0k})\right)\right]\right], \quad (1)$$

with variance

$$\mathrm{var}(B) = \frac{2\sigma_K^2}{N_K}\left[1 - \sum_{k=1}^{K-1}\left(\rho_{kK}^2\frac{N_k - N_K}{N_k}\right) + \sum_{k=1}^{K-2}\sum_{k'=k+1}^{K-1} 2\rho_{kK}\rho_{k'K}\rho_{kk'} \left(min(N_k, N_{k'})\frac{N_K}{N_k N_{k'}} + 1 - \frac{N_K}{N_k} - \frac{N_K}{N_{k'}}\right)\right]. \quad (2)$$

Estimates $\hat{B}$ and var$(\hat{B})$ follow from estimates of the correlations $\rho_{kk'}$, and standard deviations σ_k and σ_K, obtained from the appropriate regression models, using all available data.

B and var(B) for two early outcomes

For a two-arm study, participants are randomized to either the control ($j = 0$) or active intervention ($j = 1$) arms, and at an interim analysis long-term (final) outcomes are available from N_{j3} subjects and early (short-term) outcomes from N_{j1} and N_{j2} subjects in each arm of the study. Denoting the final outcome data for study participant i by X_{ij3}, and early outcomes by X_{ij1} and X_{ij2}, then following Galbraith and Marschner [1], the treatment effect B, which uses all the available early endpoint data, is given by

$$B = \eta_3 + \gamma_{13}\eta_1 + \gamma_{23}\eta_2,$$

where

$$\eta_3 = \frac{1}{N_{13}}\sum_{i=1}^{N_{13}} X_{i13} - \frac{1}{N_{03}}\sum_{i=1}^{N_{03}} X_{i03},$$

$$\eta_2 = \frac{1}{N_{13}}\sum_{i=N_{13}+1}^{N_{12}} X_{i12} - \frac{1}{N_{03}}\sum_{i=N_{03}+1}^{N_{02}} X_{i02} - \frac{N_{12}-N_{13}}{N_{13}}\left\{\frac{1}{N_{12}}\sum_{i=1}^{N_{12}} X_{i12}\right\} + \frac{N_{02}-N_{03}}{N_{03}}\left\{\frac{1}{N_{02}}\sum_{i=1}^{N_{02}} X_{i02}\right\},$$

$$\eta_1 = \frac{1}{N_{13}}\sum_{i=N_{13}+1}^{N_{12}} X_{i11} - \frac{1}{N_{03}}\sum_{i=N_{03}+1}^{N_{02}} X_{i01} - \frac{N_{11}-N_{13}}{N_{13}}\left\{\frac{1}{N_{11}}\sum_{i=1}^{N_{11}} X_{i11}\right\} + \frac{N_{01}-N_{03}}{N_{03}}\left\{\frac{1}{N_{01}}\sum_{i=1}^{N_{01}} X_{i01}\right\},$$

and γ_{13} and γ_{23} are the regressions of X_3 on X_1 and X_2 respectively, adjusted for the intervention effect. The variance of B is given by

$$\text{var}(B) = \frac{\sigma_3^2(N_{03}+N_{13})}{N_{03}N_{13}}\left[1 - \rho_{13}^2\frac{N_{01}+N_{11}-N_{03}-N_{13}}{N_{01}+N_{11}} - \rho_{23}^2\frac{N_{02}+N_{12}-N_{03}-N_{13}}{N_{02}+N_{12}} + 2\rho_{13}\rho_{23}\rho_{12}\left(1 - \frac{N_{03}+N_{13}}{N_{02}+N_{12}}\right)\right].$$

Estimates $\hat{B}$ and $\text{var}(\hat{B})$ follow from estimates of the correlations ρ_{13}, ρ_{23} and ρ_{12}, regression coefficients γ_{13} and γ_{23} and standard deviations, σ_1, σ_2 and σ_3, obtained from the appropriate regression models, using all available data.

R code for worked example

The below code implements the worked example in the main text. Before running the `gsDesign` [2] package should be installed in R [3].

```
# planned early looks
N <- 30; N.1 <- c(20, 25); N.2 <- c(15, 20); N.3 <- c(10, 15)
# expected information
information <- function(N1, N2, N3, sigma.3 = 18, rho.1.2 = 0,
rho.1.3 = 0.5, rho.2.3 = 0.5){
var <- (2 * (sigma.3 ^ 2) / N3) * (1 - (rho.1.3 ^ 2) * ((N1 - N3)/N1) -
(rho.2.3 ^ 2) * ((N2 - N3)/N2) +
2 * rho.1.2 * rho.1.3 * rho.2.3 * (1 - N3/N2))
return(1/var)
}
# at look 1
i.look.1 <- information(N1 = N.1[1], N2 = N.2[1], N3 = N.3[1])
# at look 2
i.look.2 <- information(N1 = N.1[2], N2 = N.2[2], N3 = N.3[2])
# at end
i.end <- information(N1 = N, N2 = N, N3 = N)
```

```
v.info <- c(i.look.1, i.look.2, i.end) / i.end
v.info

# calculate boundaries
library(gsDesign)
# set alphas
alpha.star.u <- c(0.000, 0.001, 0.025)
alpha.star.l <- c(0.020, 0.600, 0.975)
# modify for entry into gsDesign
gs.alpha.star.u <- diff(c(0, alpha.star.u))
gs.alpha.star.l <- diff(c(0, alpha.star.l))
gs.bound <- gsBound(I = v.info, trueneg = gs.alpha.star.l,
falsepos = gs.alpha.star.u)
bounds <- rbind(lower = gs.bound$a, upper = gs.bound$b)
bounds

# data
study.data <- data.frame(X.1 = c(56,65,59,40,43,88,34,40,49,62,42,
49,54,76,54,30,52,57,52,70,50,57,39,41,19,82,82,71,37,68,64,47,53,
51,69,37,48,57,88,60,89,53,56,60,71,32,63,84,25,32,69,26,64,59,58,
46,28,37,71,64), X.2 = c(71,70,16,80,78,78,78,55,44,95,53,48,68,62,
70,28,57,52,91,52,67,50,73,55,59,62,67,63,34,82,60,53,58,46,64,73,
69,72,63,74,72,61,93,62,43,68,66,65,62,40,47,60,54,63,74,42,24,60,
100,62), X.3 = c(64,93,86,91,83,100,82,59,65,91,70,76,86,90,80,56,
88,81,64,48,80,80,100,86,78,84,82,91,73,72,78,41,59,84,100,49,82,
74,66,79,92,83,88,76,77,67,86,72,66,60,77,52,66,86,75,65,29,48,85,
97), treat = rep(c(0, 1), each = 30))

# calculate test statistic
# change look to 2 to get calculation at second look
look <- 1
# sample sizes at look
N1 <- N.1[look]; N2 <- N.2[look]; N3 <- N.3[look]

# effect estimates
eff.X <- function(x, xn, xvar, N3){
s.X <- c(x[x$treat == 0, xvar][1:xn], x[x$treat == 1, xvar][1:xn])
treat.X <- rep(seq(0, 1), rep(xn, 2))
reg.X <- lm(s.X ~ factor(treat.X))
sigma.X <- summary(reg.X)$sigma
if(xvar == "X.1" | xvar == "X.2"){
eff.X  <- sum(x[x$treat == 1, xvar][(N3 + 1):xn] -
x[x$treat == 0, xvar][(N3 + 1):xn]
- rep(reg.X$coef[2], (xn - N3)))} else {
eff.X <- as.numeric(reg.X$coef[2])
}
return(list(eff.X = eff.X, sigma.X = sigma.X))
}
eff.2 <- eff.X(x = study.data, xn = N2, xvar = "X.2", N3 = N3)
eff.1 <- eff.X(x = study.data, xn = N1, xvar = "X.1", N3 = N3)
eff.3 <- eff.X(x = study.data, xn = N3, xvar = "X.3", N3 = N3)

# regressions
reg.XY <- function(x, xvar = "X.1", yvar = "X.3", yn = N3){
X <- c(x[x$treat == 0, xvar][1:yn], x[x$treat == 1, xvar][1:yn])
Y <- c(x[x$treat == 0, yvar][1:yn], x[x$treat == 1, yvar][1:yn])
treat.Y <- rep(seq(0, 1), rep(yn, 2))
reg.X.Y <- lm(Y ~ factor(treat.Y) + X)
gamma.X.Y <- coef(reg.X.Y)[3]
return(as.numeric(gamma.X.Y))
}
gamma.1.3 <- reg.XY(x = study.data, xvar = "X.1", yvar = "X.3", yn = N3)
gamma.2.3 <- reg.XY(x = study.data, xvar = "X.2", yvar = "X.3", yn = N3)
gamma.1.2 <- reg.XY(x = study.data, xvar = "X.1", yvar = "X.2", yn = N2)
# full model
get.subdata <- function(x, yn){
sdata <- rbind(x[x$treat == 0,][1:yn,], x[x$treat == 1,][1:yn,])
return(sdata)
}
reg.1.2.3 <- lm(X.3 ~ factor(treat) + X.1 + X.2,
data = get.subdata(x = study.data, yn = N3))
```

```
# estimate B
B.hat <- eff.3$eff.X + (1 / N3) * eff.1$eff.X * gamma.1.3 +
(1 / N3) * eff.2$eff.X * gamma.2.3

# estimate correlations
C <- matrix(c(gamma.1.3 * (eff.1$sigma ^ 2),
gamma.2.3 * (eff.2$sigma ^ 2)), ncol = 2)
D <- matrix(c((eff.1$sigma ^ 2), gamma.1.2 * (eff.1$sigma ^ 2),
gamma.1.2 * (eff.1$sigma ^ 2), (eff.2$sigma ^ 2)), ncol = 2)
sigma.3 <- sqrt(as.numeric(summary(reg.1.2.3)$sigma ^ 2 +
C %*% solve(D) %*% t(C)))
rho.1.3 <- as.numeric(gamma.1.3 * eff.1$sigma / sigma.3)
rho.2.3 <- as.numeric(gamma.2.3 * eff.2$sigma / sigma.3)
rho.1.2 <- gamma.1.2 * eff.1$sigma / eff.2$sigma

# estimate variance
info.look <- information(N1 = N1, N2 = N2 , N3 = N3, sigma.3 = sigma.3,
rho.1.2 = rho.1.2, rho.1.3 = rho.1.3, rho.2.3 = rho.2.3)
vB.hat <- 1/info.look

# test statistic
z <- B.hat/sqrt(vB.hat)
z

# decision making
if(z < bounds["lower", look]){decision <- "STOP: Futility"}
if(z > bounds["upper", look]){decision <- "STOP: Efficacy"}
if(z > bounds["lower", look] &
z < bounds["upper", look]){decision <- "CONTINUE"}
decision
```

Author details

[1] Statistics and Epidemiology Unit, Warwick Medical School, University of Warwick, CV47AL Coventry, UK. [2] Warwick Clinical Trials Unit, Warwick Medical School, University of Warwick, CV47AL Coventry, UK. [3] University Hospital Coventry and Warwickshire, CV2 2DX Coventry, UK.

References

1. Galbraith, S., Marschner, I.C.: Interim analysis of continuous long-term endpoints in clinical trials with longitudinal outcomes. Stat Med **22**(11), 1787–805 (2003)
2. Anderson, K.: gsDesign: Group Sequential Design. (2016). R package version 3.0-1. https://CRAN.R-project.org/package=gsDesign
3. R Core Team: R: A Language and Environment for Statistical Computing. R Foundation for Statistical Computing, Vienna, Austria (2018). R Foundation for Statistical Computing. https://www.R-project.org/
